# Female Fertility Preservation, Clinical and Experimental Options

**Published:** 2018

**Authors:** Zahra Rajabi, Fereshteh Aliakbari, Hossein Yazdekhasti

**Affiliations:** 1- Department of Biomedical Engineering, University of Virginia, Virginia, USA; 2- Department of Anatomy, School of Medicine, Tehran University of Medical Sciences, Tehran, Iran; 3- Infertility and Reproductive Health Research Center, Shahid Beheshti University of Medical Sciences, Tehran, Iran; 4- Department of Molecular Physiology and Biological Physics, Center for Membrane and Cell Physiology, University of Virginia, Virginia, USA

**Keywords:** Cancer treatment, Cryopreservation, Embryo, Fertility preservation, Freezing, Oocyte, Ovarian tissue, Transplantation

## Abstract

Recently, due to tremendous progress in prognosis, diagnosis, and treatment of different kinds of malignancies, demands on fertility preservation were raised significantly in developed countries. Fertility failure is one of the most detrimental consequences of radio/cytotoxic treatment procedures in women who could overcome their cancer disease. For women who are involved in cancer diseases, there are multiple options regarding their fertility preservation and those could be selected according to patient’s age, the risk of ovarian involvement, the available time and the type of cancer with different levels of advantages and disadvantages. Although there are multiple options, but embryo cryopreservation and ovarian tissue cryopreservation are the most reliable methods for permature and post-mature puberty, respectively. In addition, other approaches like artificial ovary, isolation and cryopreservation of follicles and mature and immature oocyte preservation are under investigations and the success rate of oocyte vitrification is increasing. Therefore, the techniques have the potential to be used in clinic in near future. The presence of comprehensive consultation, before the onset of any kind of cancer treatment procedures, is an indispensable issue which would help patients to make up their mind in choosing the immediate and the best available fertility preservation option.

## Introduction

The incidence rate of cancer among reproductive-age women is 7%, and current cancer treatment methods and strategies have increased survival rate five years following cancer treatment (1, 2). One of the major concerns for women with a history of cancer is infertility issue after malignancy treatments (3). Recently, due to early detection of cancer procedures and tremendous progress that has been made in fertility preservation and dramatic increase of 5 years in survival rate following cancer treatment (4), the demands for fertility preservation in young cancer patients significantly increased (5). Fertility failure is one of the most detrimental consequences of radio/cytotoxic treatment procedures in women who could overcome their cancer disease. Chemotherapy, particularly with alkylating agents such as busulphan, ionizing radiotherapy in the abdomen or pelvic region, gynecological malignancies surgery, can permanently destroy gonads and lead to infertility and premature menopause (6, 7). Therefore, development of fertility preservation strategies helps the patients in having adequate options before experiencing aggressive treatments. Among them, patients with certain benign conditions like autoimmune and hematologic conditions, the presence of bilateral ovarian tumors, severe or recurrent ovarian endometriosis and recurrent ovarian torsion may mostly benefit from the development of such strategies (8, 9).

The American Society of Clinical Oncology (ASCO) has recently updated its guidelines for fertility preservation in cancer patients according to same pregnancy rate results obtained from both oocyte and embryo cryopreservation which were reported by American Society for Reproductive Medicine (ASRM) (10). Ovarian transposition and embryo cryopreservation for pubertal female patients were two most common and appropriate fertility preservation strategies before 2013. However, in the current revised guideline of ASCO, oocyte cryopreservation has been recommended as another applicable option for fertility preservation in females at pubertal stage (10). In addition, ovarian cryopreservation is another approach which has a great potential to be an option for cancer patients in the near future. Meanwhile, newly developed promising strategies like *In Vitro* activation (IVA) of primordial follicles have increased the hopes and allow premature ovarian failure (POF) and primary ovarian insufficiency (POI) patients to conceive using their own eggs (11, 12).

**Figure 1.**
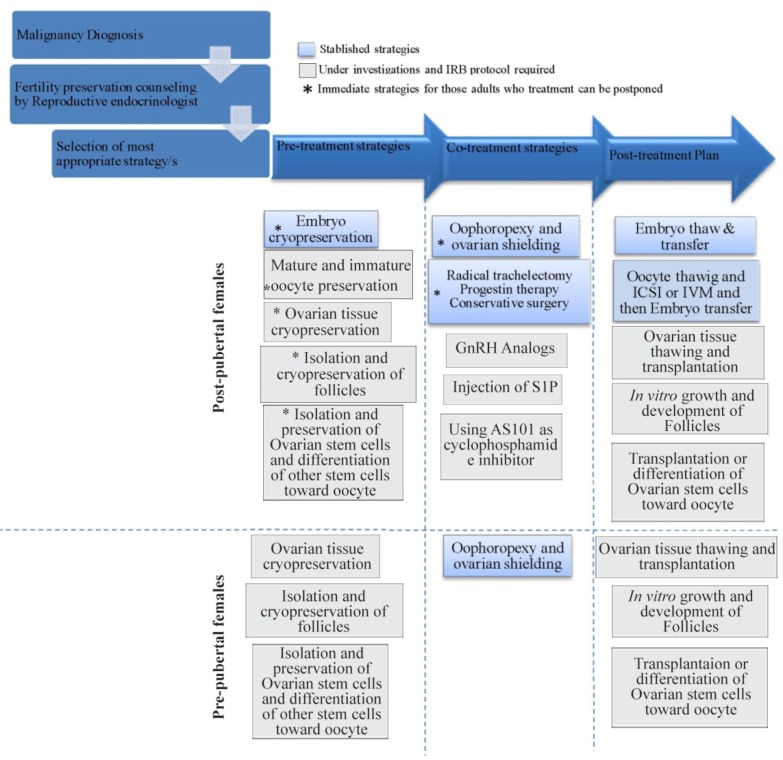
Recommended fertility preservation approaches in women

There are different strategies for fertility preservation which can be applied based on patient’s age and status as well as the risk of ovarian involvement (13). Some of these strategies showed their sufficient efficiency, and they are now a part of routine procedures of ART clinics, while others are still under investigation. Also, there are some other conditions in which non-oncological patients may benefit from fertility preservation strategies (14, 15). For example, some patients post-pone their parenthood into their fourth and fifth decade of life due to different reasons such as economic situation and industrialization and other conditions in which they are treated with gonadotoxic agents that can lead to premature ovarian failure including some chromosomal abnormalities (Turner’s syndrome) and individuals with severe or recurrent endometriosis as well as ones with autoimmune disorders (14). These strategies are a combination of recent advances in assisted reproductive technologies (ARTs), cryotechnologies, and novel cell culture systems. In this review, existing fertility preservation strategies besides their advantages and disadvantages were briefly explained and compared, and for a better explanation of each method and its applicability, they were categorized according to the pubertal status of patients (Figure 1).

### Adult female patients and fertility preservation options:

Most of the possible strategies for fertility preservation in pubertal patients are costly and invasive, and none of them are as reliable as sperm banking in men. There are multiple strategies with different levels of efficiency, applicability, advantages, and disadvantages regarding fertility preservation among adult women at ART centers (Table 1), wherein the recruitment of best options directly depends on the type of cancer, patient’s age, the available time, and whether the likelihood of ovarian involvement is high.

**Table 1.** Advantages and disadvantages of each fertility preservation approaches in women

**Method**	**Advantages**	**Disadvantages**
**Embryo cryopreservation**	Well-established and the most reliable option Higher pregnancy rate Best option when enough time is available before onset of treatment	Non-applicable for prepubertal girls Requires a male partner or sperm donation Requires ovarian stimulation Takes 2–5 weeks Arising some ethical concerns Non-applicable for women who have hormone-sensitive cancers Expensive method Outpatient surgical procedure
**Mature oocyte cryopreservation**	Applicable for singles and those who are not pleasant to have sperm donation No ethical problems No urgent need for sperm	Low pregnancy rate, but acceptable fertilization rate Still needs ovarian stimulation and delay in cancer treatment At least 20 vitrified oocytes are required to achieve a live birth Not suitable for those women with hormone-sensitive cancers Not suitable for PCOS patients due to high risk of OHSS Expensive method Outpatient surgical procedure
**Immature oocyte cryopreservation**	No delay in cancer treatment Higher fertilization rate in comparison with mature oocyte No ovarian stimulation is needed No risk of OHSS in PCOS patients Can be performed during ovarian tissue cryobanking or oophorectomy No ethical problems No urgent need for sperm Not expensive Abundantly available germ cells Lack of spindle and zona pellucida and less damage during freezing	Has not been developed in patients with cancer IVM is needed
**Ovarian tissue cryopreservation**	The only available approach for prepubertal girls and women who cannot delay the start of chemotherapy Allows natural pregnancy after autotransplantation Carries fewer ethical dilemmas No ovarian stimulation is needed There is no need for partner or sperm donation	Low success rate Some complications with activation of primordial follicle following thawing Some safety and ethical issues following xenotransplantation Not suitable if the risk of ovarian involvement is high Not recommended to be performed during controlled ovarian stimulation Not available in every clinical center There is also the risk of ovarian failure after removing an ovarian volume Potential risk of cancer recurrence IVF needed following xenotransplantation Multiple practical procedures are required There are so many unsolved issues in human ovarian transplantation
**Oophoropexy**	Can be performed for all ages Oophoropexy by laparoscopy is simple, safe, and effective Can be performed immediately before pelvic irradiation Ovarian function can be preserved No ethical problems	No satisfying results Risk of other complications Spontaneous pregnancy may not be possible A second procedure is needed to relocate ovaries back to pelvis for spontaneous pregnancy Oocyte retrieval may become technically more challenging Some treatment delay is needed for healing incision
**GnRH Analogs**	No surgery is needed Not expensive and easy to perform Spontaneous pregnancy is possible Ovarian function can be preserved	Needs some more evidence to be applicable

### Embryo cryopreservation:

Cryopreservation of embryo is the most relevant and well-established option for fertility preservation and was the only method endorsed by the ASRM until 2012. The effectiveness and safety of this option have been proved so that this technique is routinely used in ART centers for infertile women to store supernumerary embryos, prevent ovarian hyperstimulation syndrome (OHSS) and in cases of impaired endometrial development and impractical embryo transfer (16, 17). Meanwhile, this should be considered that hyperstimulation regimes, especially estrogen, lead to growth of some hormone-dependent malignancies and some other complications may occur during oocyte retrieval including hemorrhage and infection in patients with low blood counts.

Although the success rate of oocyte vitrification is increasing, embryo cryopreservation, based on obtained satisfactory rate, is still the best option that can be offered to post-pubertal women who desire fertility preservation, especially for those who are mature and have enough time prior to onset of aggressive treatments and have a partner or sperm donor as well. This is noteworthy to mention that transfer of cryopreserved embryos leads to improvement of pregnancy rate compared to fresh embryo transfer strategy, due to improved embryo–endometrium synchrony (18).

### Mature or immature oocyte cryopreservation:

Cryopreservation of oocyte is another option especially for those women who are single and are not pleasant to have sperm donation. Recent reports are showing that the success rate and efficiency of oocyte vitrification significantly have improved and are comparable with obtained results from fresh oocytes (19, 20). In addition, previous reports indicated that this technique has an overall survival rate of 92.5% and an ongoing pregnancy rate of 43.7% in egg donation programs (21). Meanwhile, there is another strategy especially to avoid any delay due to hyperstimulation, wherein immature oocyte can be retrieved during ovarian tissue cryobanking or oophorectomy (22). Some researchers believe that immature oocytes, metaphase I or germinal vesicle oocytes, are less vulnerable to harmful effects of cooling and cryodamages due to lower cell volume, and lack of metaphase spindle. However, the rate of developmental capacity and pregnancy is low, although the rate of nuclear maturation is still high in the cryopreserved immature oocyte (23, 24). Nevertheless, no similar live births have been reported in cancer patients, at least based on our knowledge, and still further investigation is needed for a routine clinical procedure.

### Ovarian tissue cryopreservation:

Another alternative for fertility preservation in women with oncological and non-oncological diseases is ovarian tissue cryopreservation (OTC) (25). Some studies have shown that immature oocytes within primordial follicles are more resistant to cryopreservation damage (26) and this method has this potential to be a suitable option. However, the main question and concern regarding this technique is how to activate quiescent follicle after freezing and thawing. There are three possible options to address this fundamental issue including ovarian tissue autotransplantation, xenotransplantation and *in vitro* culture system. In autotransplantation, that is the only clinically applicable strategy at recent, ovarian tissues can be returned to the patient by autotransplantation (Either orthotopically or heterotopically) (27), which would allow natural fertility, but *in vitro* fertilization still remains an option in case previous methods failed. Although, many investigations have been done to improve OTC efficiency, but the number of live births by this method is still meager, and further investigations are needed to establish this technique as an available option in ART centers, particularly on improvement of revascularization process with the aim of reducing the follicular loss which occurs after tissue grafting (28). The xenotransplantation approach is still a promising method but is problematic because of safety and ethical issues. Recently, huge progress has been made in the culture of immature follicles, mainly using three-dimensional culture strategies (29, 30), which allows overcoming obstacles that exist within the culture of human follicles *in vitro*. Recently, a new infertility treatment method has been developed called “*In vitro* activation (IVA)” which allows POF patients to conceive using their own eggs via activation of residual dormant follicles. Multiple pregnancies have been reported using this strategy wherein stimulation of phosphatidylinositol-3-kinase (PI3K) and AKT-forkhead box O3 (FOXO3) pathways followed by disruption of Hippo signaling pathway using ovarian fragmentation, leads to activation of primordial follicle growth and subsequently, ovarian tissue autotransplantation may help to achieve successful pregnancies (11). Also, in another recent achivment, patients with POI underwent the same procedure starting by ovarian vitrification which led to reported successful follicle growth and pregnancies (12).

Based on recent reports, almost 100 children were born or will be born in the near future using OTC. All gestational age and birth weight in these newborns were within internationally recognized normal standards and it can be suggected that frozen-thawed ovarian tissue transplantations is going to be a routine fertility preservation (31).

### Oophoropexy:

Oophoropexy is an applicable method which is routinely used in clinics for Hodgkin disease and other patients for whom abdominal surgery is necessary before irradiation, in order to minimize harmful effects of ionizing irradiation on ovarian function (32). In this procedure, ovaries are transposed either to under the uterus or out of radiation field and the technique can be immediately performed before onset of scheduled radiotherapy through laparoscopic section (33). Due to direct dependency of fertility preservation results by this method to different variables such as patient’s age, radiation dose, shielded ovaries, use of concomitant chemotherapy (7, 34), in this approach, the efficiency and obtained results are not satisfying and there is risk of other complications which may occur (35).

### Gonadotropin-releasing hormone (GnRH) analogs:

In one clinical trial, it was indicated that 94% of treated patients with GnRH agonist showed initiation of spontaneous menstruation within 3–8 months after termination of chemotherapy demonstrating that GnRH agonist can prevent the ovaries from being damaged during chemotherapy (36). Although there is no protective effect of GnRH analogs for primordial follicles from radiation therapy, it has been shown that primordial follicles could be protected from cytotoxic cancer therapy procedures but, its clinical applicability is still controversial (37–39). Protective effects of GnRHa on ovarian function during chemotherapy have been proposed due to granulosa cells mitotic activity reduction following the administration of GnRHa (40) and the inhibition of recruitment process of mice preantral follicles and their growth and development to antral follicles.

Some other promising methods have been recently developed, wherein devastating consequences of radio/cytotoxic treatment procedures are diminished by suppressing apoptosis pathways in the germ cells. Sphingosine-1-Phosphate (S1P) (41), AS101 (42) and imatinib (43) are examples of such approaches and their future applicabilities require further investigations and clinical trials.

### Pediatric and adolescent female patients and fertility preservation options:

There are multiple possible options that might be helpful for prepubertal girls to preserve their fertility before the onset of aggressive cancer treatment. Among them, the most applicable and valid option for adolescent female patients is OTC that is considered as an experimental method yet and needs some more investigations and approval by Institutional Review Board (IRB) (10). In the last decay, extensive attempts have been done to successfully crypreserve ovarian tissue in prepubertal girl, but unfortunatley, no live births have been reported yet from ovarian tissue cryopreservation and transplantation from a prepubertal girl. Beside this, there is high risk of reintroduction of original disease after transplantation in these cases indicating the necessity of IVM and artificial ovary methods improvement (9, 44).

Isolation of follicles from ovarian biopsies and cortical strips is another approach for young patients (45). Even though this approach was partially successful in mice, however, this requires further studies to be completely applicable for patients who suffer from cancer disease. In this method, multiple ovarian biopsies are removed from young patients through either laparoscopy section or oophorectomy and then follicles can be isolated from ovarian tissue and cryopreserved for a long time and following completion of cancer treatment, follicles can be either returned to patients via autotransplantation or thawed and cultured and then matured *in vitro* to produce off-spring by *in vitro* fertilization. This is worthy to mention that shielding ovaries or oophoropexy is another possible approach, wherein ovaries can be removed from irritation field (46, 47). However, irritation has detrimental effects on the function of the uterus so that the probability of natural pregnancy is significantly reduced following pelvic irradiation (48), although ovarian function can be preserved by this way.

### Future perspectives:

In a newly developed strategy called “transplantable artificial ovary”, for eliminating the the risk of transmission of malignant cells, the isolated primordial follicles are transferred into matrices, mostly using fibrin, and then grafted to the inner part of the peritoneum to create an artificial organ. Advances in isolation and washing procedures prior to encapsulation of primordial follicles have led to impressive achivments in this approach and in some studies resulted in growing of human antral follicles after autografting primordial follicles inside a fibrin scaffold in a mouse model (49, 50).

Recently, another likely option has been proposed, wherein putative ovarian stem cells (Or stem cells from other sources) can be isolated from patient’s ovarian tissues and be differentiated toward competent oocyte either by injecting back into ovarian tissue *in vivo* or in *in vitro* culture (51–53). This option is still in its infant stage, and in order to overcome existing controversies in this area, further investigations are needed (54–56).

## Conclusion

Current advances and successful reported pregnancies using ovarian cryopreservation and transplantation and artificial ovary and also utilization of multistep culture system of primordial follicles as well as allografting option, have increased hopes in cancer survivors. Following transplantation, follicles loss is one of the results of non-efficient revascularization of the graft and tissue ischemia; however, these consequences could be prevented by using S1P, Vascular Endothelial Growth Factor (VEGF), Anti Mullerian Hormone (AMH), antioxidants and innovative stem cells therapy approaches (22, 57–61). Regardless of methods mentioned above, IVF using donor oocyte is another approach which is still an option for cancer survivors with diminished ovarian reserve (62). This is definitely vivid that women with cancer should benefit from adequate and useful consultations regarding their fertility preservation options and immediate and correct option selection according to updated guidelines. Therefore, the probability of childbearing after aggressive cancer treatments can be increased.
